# “Irritable Hip”: Diagnosis in the Emergency Department. A Descriptive Study Over One Year

**DOI:** 10.7759/cureus.3481

**Published:** 2018-10-23

**Authors:** Ahmer Irfan, Robert J Starr, Steven Foster, Innes D Smith, James S Huntley

**Affiliations:** 1 Department of Surgery, Johns Hopkins Hospital, Baltimore, USA; 2 Department of Anaesthesiology, Aberdeen Royal Infirmary, Aberdeen, GBR; 3 Department of Paediatric Emergency Medicine, Royal Hospital for Children, Glasgow, GBR; 4 Department of Trauma and Orthopaedics, Queen Elizabeth University Hospital, Glasgow, GBR; 5 Department of Surgery, Sidra Medicine, Ar-Rayyan, QAT

**Keywords:** transient synovitis, irritable hip, epidemiology, kingella kingae

## Abstract

Background

A ‘limping child’ commonly presents to the emergency department (ED), often without a history of trauma. It is important that serious underlying pathology is ruled out before a diagnosis of benign irritable hip (IH). The aetiology of IH is not well understood and there may be geographical and seasonal variation. The aim of this study was to determine the basic epidemiology of IH in the Glasgow Population.

Methods

A retrospective analysis was carried out of all children discharged from the Glasgow Children’s Emergency Department from January to December 2016. Relevant discharge codes were determined and patient records screened. Any patient who did not have a discharge code had their presenting complaint and medical record screened.

Results

A total of 354 patients were diagnosed with IH, of which 319 and 189 were in the Greater Glasgow and Clyde and City of Glasgow catchment areas, respectively. The majority of these patients (n = 254) were diagnosed clinically. The incidence of IH was 177.7 per 100,000 children with a boy:girl ratio of 1.9:1 (209:110). The mean age of presentation was 3.5 years and the recurrence rate was 5.9% (n = 18). There was an increased incidence in spring (n = 111), especially in March (n = 42) and April (n = 40). There was no incidence variation or influence discernible by social deprivation.

Conclusion

In this population, IH has: (i) an atypical age profile (age distribution shift to younger), (ii) no marked association with social deprivation (in contrast to other studies), and (iii) a 'spring preponderance'. We suggest that most cases can safely be managed in the ED without recourse to further investigations or speciality referral.

## Introduction

A ‘limping child’ commonly presents to the emergency department (ED), often without a preceding history of trauma [1]. It is important that serious underlying pathology is not missed, including septic arthritis (SA), osteomyelitis, bone tumors, leukaemia, Perthes disease and slipped capital femoral epiphysis (SCFE) [2]. Once these are ruled out through a combination of history, examination and/or investigations, a diagnosis of benign irritable hip (IH) can be made, and the patient managed conservatively.

The most common cause of IH is transient synovitis (TS), pathologically defined by synovial inflammation and associated effusion, and diagnosed by ultrasound [3]. However, children can present with features of IH but negative ultrasound [1]. These two conditions, TS and IH with negative ultrasound, can be grouped under the term of benign IH.

The self-limited nature of this condition is a key feature, with resolution occurring within 14 days [4]. Persistence beyond this time should raise the suspicion of a more serious aetiology [5]. Recurrence of TS (including on the contralateral side) is not uncommon [6,7] but case series do not suggest that recurrence leads to a poorer prognosis [6-8]. It was debated whether TS might increase the risk of Perthes disease. However, patients diagnosed with Perthes had persistent symptomatology [9], which would not be consistent with a diagnosis of TS – rather than TS causing Perthes, it is likely that early Perthes is simply difficult to differentiate from benign IH.

Although a limp is a common presenting complaint for IH, other symptoms include pain (which can often be poorly localised in the limb) [1], restricted range of movement [10] and/or a low grade fever [11].

The epidemiological data for this condition are predominantly European. However, the aetiology of benign IH is not well understood and there may be geographical variation. Several theories have been proposed for the underlying aetiology. A post-infective state is the leading hypothesis, as an association has been shown between TS and preceding viral infections [12]. However, no particular infective agent has been identified. It was believed that such an association would lead to a seasonal variation in IH/TS, but this hypothesis is still disputed [3,13]. The mean age of presentation was 5.9 years with a preponderance in boys [14].

The aim of this study was to determine the basic epidemiology of paediatric IH in the Glasgow population. Unfortunately, the terms ‘irritable hip’ and ‘transient synovitis’ are often used interchangeably in primary care and the ED. For our purposes, the term IH is used for any benign diagnosis of hip irritability for which no serious underlying pathology was found.

## Materials and methods

Hospital and locale

The Glasgow 2011 Census documented the age and ethnicity of children in the Greater Glasgow and Clyde (GG&C) Health Board. This was used to determine incidence of disease in the population. The total population of children aged 0–14 years in the GG&C area was 179,448. The Royal Children’s Hospital in Glasgow is the only Children’s ED in the City of Glasgow (CoG) and the tertiary referral centre for the West of Scotland. This geographic restriction implies majority case-capture for children, although many patients with IH could still be treated or observed in primary care. An implicit assumption was made that the population of people aged 0–14 years has remained static from the time of census to present.

Retrospective analysis and search methodology

**Figure 1:**
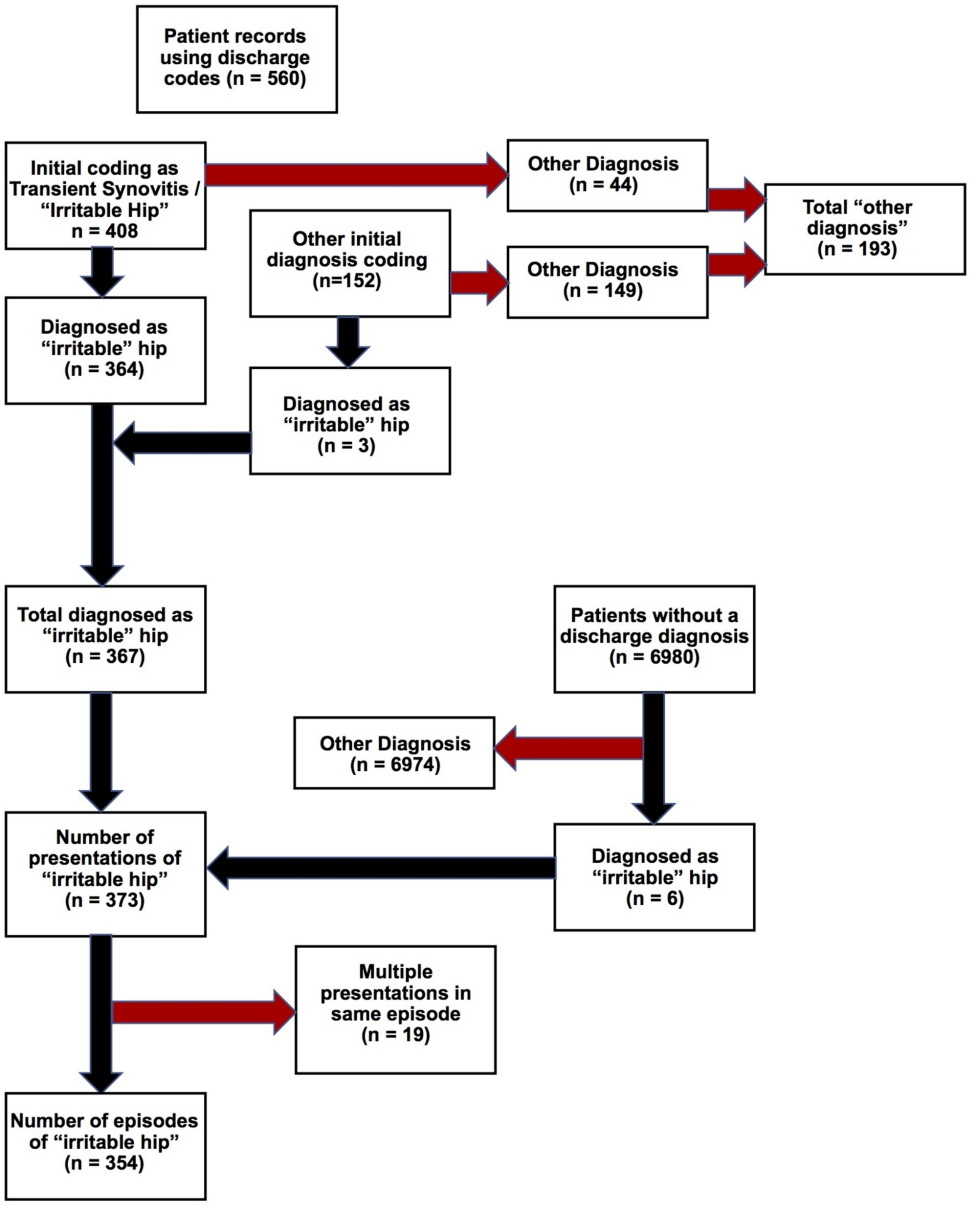
Flowchart demonstrating search methodology.

The data were collected in January 2018 and concerned all patients presenting to the Royal Hospital for Children (Glasgow) Emergency Department (ED) over a 12-month period, 1st January to 31st December 2016. On discharge from the ED at initial visit, patient episodes were coded on Trakcare (InterSystems TrakCare®) based on their ED diagnosis. Notes with all potentially relevant discharge codes were screened (Appendix 1) (n = 560) to determine final diagnosis if available. Within this cohort, 408 patients had a discharge diagnosis of transient synovitis/irritable hip and 152 patients had other discharge diagnoses from those screened. The presenting complaint and records of all patients who did not have an ED discharge code, were also screened during this time frame (n = 6980) to ensure no cases were missed (Figure 1). These patients had had a full ED workup and diagnosis but no final discharge letter was written, and subsequently a final discharge code had not been entered into the system.

Inclusion/exclusion criteria

Children were included up to the age of 14 years at the time of presentation. All patients who were diagnosed as having IH based on clinical examination, investigations or both were included. They were excluded if an alternative diagnosis was determined during follow-up, after their ED discharge. Any patient who had multiple presentations during the same episode (within 14 days of initial presentation) had these presentations excluded with only the initial presentation included in the final analysis (Figure 1). During analysis, some children were excluded based on geographical location of home address as they fell outwith the catchment area of the measured population (n = 35).

Social deprivation

Social deprivation was assessed using the Scottish Index of Multiple Deprivation 2016 (SIMD). The SIMD is a national deprivation score allocated by the Scottish government ranking areas from 1 (most deprived) to 6976 (least deprived). Each area was divided to contain similar population sizes (760 people per area). Deprivation was determined using 38 markers of deprivation, grouped into seven domains which considered (1) income, (2) employment, (3) education, (4) health, (5) access to services, (6) crime and (7) housing. The total population and working population for all of the ranking areas in the CoG was obtained to determine incidence of disease.

Seasonality

Season of presentation was considered using the Northern Meteorological Definition [15] which grouped the seasons into Spring (March to May), Summer (June to August), Autumn (September to November) and Winter (December to February).

Recurrence

Any patient who was re-diagnosed with IH greater than 14 days after the initial presentation (15th January 2016 to 31st December 2017) was coded as a recurrence.

Ethnicity

Patient ethnicity was self-identified and subsequently stored in their medical data on Trakcare. Patients did not have to reveal their ethnicity and therefore some of the patient records did not have this recorded (n = 77).

Statistical analysis

Where appropriate, data were analysed for statistical significance using SPSS (IBM SPSS Statistics, IBM Analytics, Armonk, NY).

## Results

Diagnosis of irritable hip

**Figure 2:**
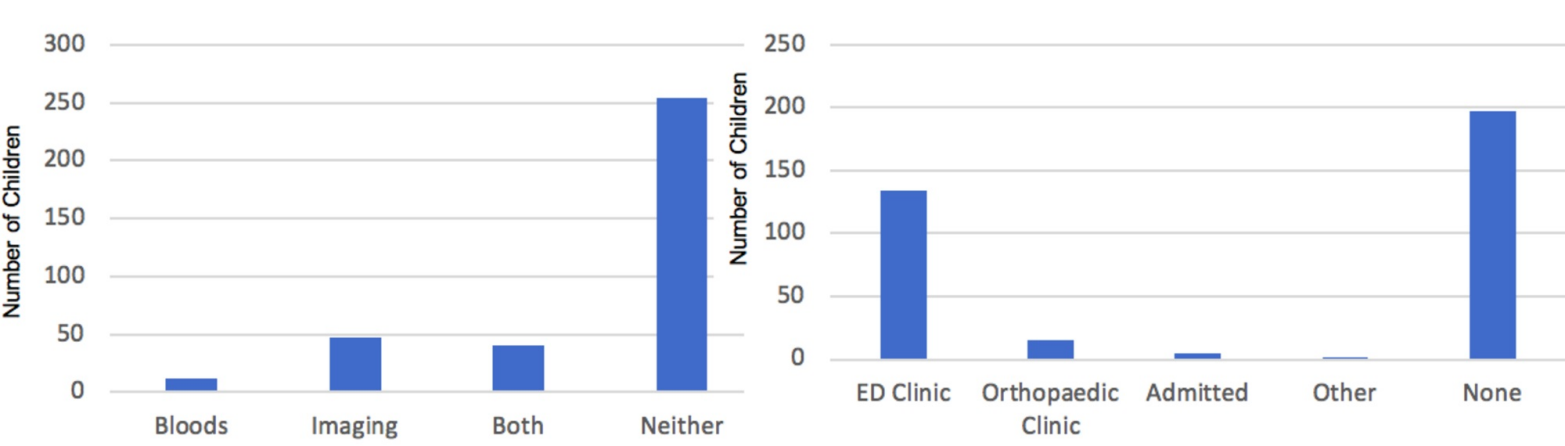
Patient investigations and follow-up attended.

IH was diagnosed in 354 patients over the one-year study period (January – December 2016). The diagnosis of IH in the Glasgow ED is primarily a clinical one. All patients who presented to the ED had a history taken and underwent an examination. The majority of patients were diagnosed as having an ‘irritable hip’ clinically (n = 254). Patients in whom the underlying diagnosis was unclear or thought to be more sinister underwent bloods alone (n = 12), imaging of the joint (n = 47) or both (n = 41) in the ED (Figure 2).

Most patients discharged from the ED with a diagnosis of IH were offered ED follow-up if symptoms did not settle, with the option of cancellation. However, the majority did not attend for follow-up (n = 197). Therefore, their non- attendance is assumed to correlate with a resolution of symptoms. The majority of patients diagnosed with IH who attended follow-up did so in the ED clinic (n = 133), usually to document absence of symptoms and to confirm the transient nature of the illness. A minority of patients were followed up in orthopaedic clinic (n = 15), admitted (n = 7) or seen in an alternative clinic (n = 2) (Figure 2).

Geographical restrictions

The Glasgow 2011 census provided population data for the GG&C health board and SIMD provided population data within the CoG council area. The postcode of each patient was screened, if they were outside of the respective health board or council area, they were removed from the incidence analysis. From the initial study population of 354 children, the number of children in the GG&C and CoG areas were 319 and 189, respectively. The GG&C population was used for analysis of overall incidence and variations due to age, sex and ethnicity. The CoG population was used for analysis of incidence variation due to social deprivation status.

Patients

The annual incidence of irritable hip in the GG&C area (n = 319) was 177.7 per 100,000 children aged 0–14 years. The incidence of irritable hip was greater in boys, with a 1.9:1 boy:girl ratio (209 boys, 110 girls, p < 0.001). There was no significant difference between the laterality of the irritable hip (165 left hips, 151 right hips, three bilateral hips). The condition recurred in 5.9% (n = 18) of patients with 11% (n = 2) on the contralateral side.

Alternative diagnoses

Of the patients that were initially coded as TS/IH, 44 were believed to have a diagnosis other than IH. These were transient synovitis in other joints (n = 12), fracture (n = 5), soft tissue injury (n = 6), Perthes disease (n = 2), juvenile idiopathic arthritis (n = 3), Osgood-Schlatter disease (n = 1), Guillain-Barre syndrome (n = 1), Langerhans cell histiocytosis (n = 1), osteomyelitis (n = 1), epiphyseal dysplasia (n = 1), non-specific abdominal pain (n = 1) and benign growing pains of childhood (n = 1). A normal examination and no diagnosis was documented for three children and six children were believed to have a diagnosis other than IH but no final diagnosis had yet been determined.

Ethnicity

In the GG&C area, 88.2% of children were identified as White Scottish and this corresponded to 69.2% of patients with irritable hip (n = 221). The incidence of irritable hip amongst all children who identified as white (n = 229) was 144.7 per 100,000 children. The next largest ethnic group identified as Asian (n = 23) with an incidence of 166.5 per 100,000 children. Due to the small population representation of other ethnic groups within the area, it is impossible to draw any conclusion regarding IH susceptibility due to ethnicity.

Seasonality

**Figure 3:**
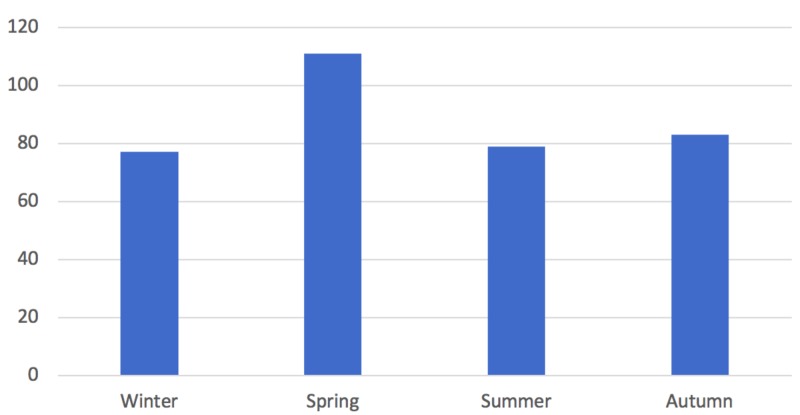
Season of incidence.

**Figure 4:**
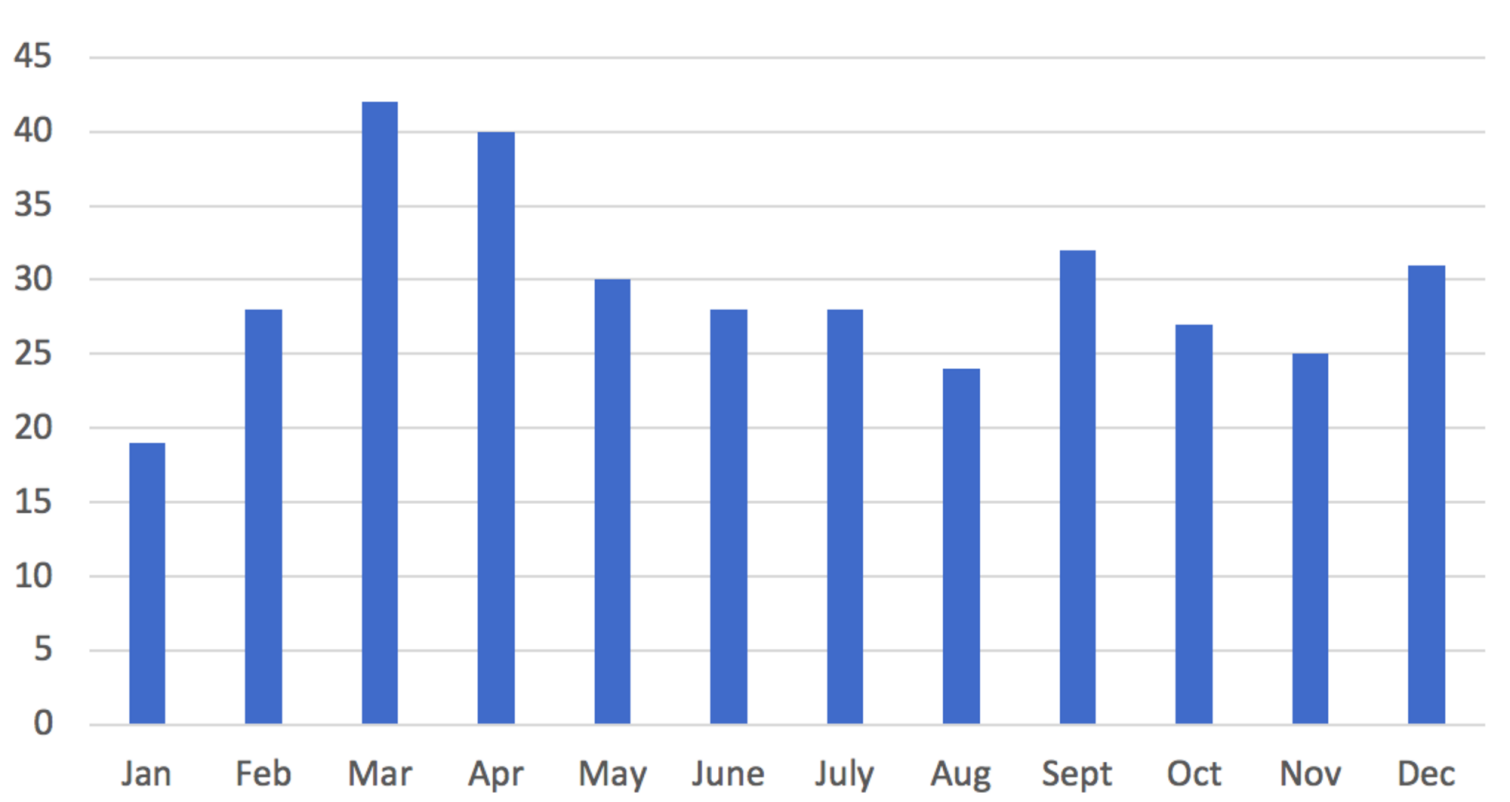
Month of incidence.

There was an increased incidence of irritable hip in spring (n = 111) when compared to the other seasons. When divided into months, this was represented with an increased incidence in March (n = 42) and April (n = 40) (Figures 3, 4).

Age at presentation

**Figure 5:**
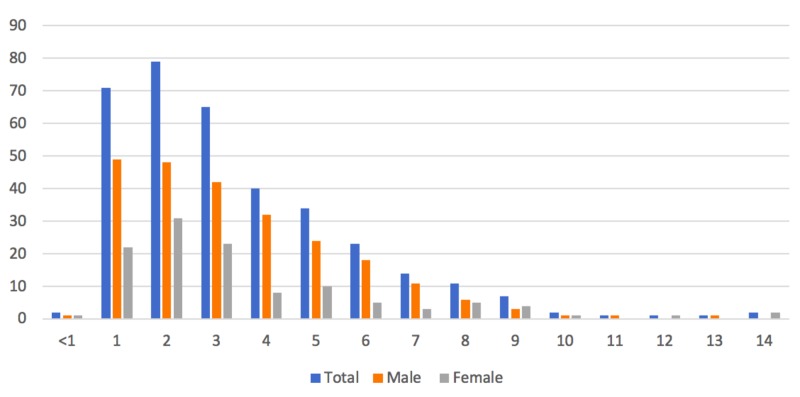
Age and gender of incidence.

For this study, it was assumed that the age distribution of children in the GG&C in the 2011 census was unchanged in the present population. Of the patients presenting with irritable hip (n = 319), the mean, median and mode age of presentation were 3.5 years, three years and two years, respectively (Figure 5).

Social deprivation

The SIMD divided Glasgow into 743 areas which were ranked as per the SIMD domains. Due to the geographical variation of the patient population, a smaller proportion (n = 189) were included in this analysis. A substantial proportion of these data zones were ranked in the 1st Quintile (most deprived), containing significantly more children (Table 1). However, there was not a significant incidence variation or trend based on SIMD Quintile.

**Table 1:** Social deprivation and incidence.

Social Deprivation Quintile	Number of Children	Non-Working Age Population	Incidence per 100,000 Children
1 (most deprived)	100	94,725	105.6
2	34	29,806	114.1
3	24	23,335	102.8
4	15	17,866	84.0
5 (least deprived)	16	16,595	96.4

## Discussion

The most common cause of IH is TS. In the literature, the incidence of benign IH can be categorised as ultrasound-positive TS or ultrasound-negative IH. Unfortunately (from a diagnostic perspective) our population did not undergo diagnostic ultrasound. The incidence of IH was 177.7 per 100,000 children. This is comparable to the incidence of ultrasound-negative IH in Malmo, Sweden: 203 per 100,000 children [13]. A study in Edinburgh, UK [1] found an incidence of 84 per 100,000 children with ultrasound-positive TS and a further 58 per 100,000 children with ultrasound-negative IH, giving a cumulative incidence of 142 per 100,000 children. The data from Merseyside, UK [3] only included patients with ultrasound-positive TS (25.1 per 100,000 children) and is therefore not a comparable data-set.

Previously, the mean age for presentation with IH has been documented as 5.9 years [14]. However, the mean age in our population was lower, at 3.5 years, with a median of two years. In our cohort, of the 354 cases of IH diagnosed in the ED in 2016, 61.6% (n = 218) were aged three or under. Generally, TS is thought to be rare in children less than three years of age – with a raised index of suspicion for an infective process, especially Kingella kingae [16]. Kingella kingae SA usually presents with less severe symptoms and investigation results, and therefore may be missed [16]. Of our cohort, only 6.9% (n = 15) had blood investigations done, with none of these children undergoing subsequent synovial fluid or blood cultures. Unfortunately, it is impossible to quantify the risk of Kingella kingae infection in our population, and this remains a clinical concern.

In the GG&C population (n = 319), the recurrence rate of IH was 5.9% (n = 18). This is lower than the recurrence rates from two case series (i) Southampton, UK: 14.8% [6] (63 out of 426 patients), and (ii) Geneva: 13.9% [7] (51 out of 366 patients). The uncertainty of the aetiology of the IH in our population may be a contributing factor for this discrepancy.

Historically, there has been a consistent male preponderance of IH with a boy:girl ratio of 2:1 [14] upon pooling the data. This is comparable to our cohort with a boy:girl ratio of 1.9:1 (209 boys, 110 girls).

In our population, there was a preponderance of irritable hips presenting in March and April. The study by Landin et al. [13] in a Scandinavian population found an October/November preponderance. Conversely, a study in Liverpool, UK [3] did not note a seasonal change. Even when taking previous historical TS case series into account, no consistent pattern has emerged for the seasonality of IH. It is not possible to categorise a snapshot single year of incidence preponderance to being attributable to seasonality per se. A season can be defined as a pattern in an outcome that increases and decreases with some regularity [17]. A consistent pattern emerging over multiple years may indicate a seasonal association. For our study, replication of multiple years of data would be required to decide if there was a consistent association with season.

Previously, there have been associations made between Perthes disease and social deprivation [18,19]. The study in Merseyside, UK [3] showed that an increase in deprivation quintile increased TS. Within our dataset, this trend is not present – with the greatest TS incidence in quintile 2 and the lowest in quintile 4. Although there can be variations in deprivation within an area, the small population size (760 people per deprivation area) may reduce this variability. As the association between IH and social deprivation has not been the subject of focus in the literature, replication in other settings would be informative.

Studies of IH epidemiology have been predominantly European with limited ethnic variation in the study populations. Ethnicity was recorded in this study but the majority of the population within our geographical restrictions identified as White Scottish (88.2%). The largest ethnic group in the Glasgow census was Asian, which comprised 2.7% of the population. The incidence of IH in Asian children (166.5 per 100,000 children) was comparable to white children (144.7 per 100,000 children). It seems that children of all ethnicities are susceptible to IH. Whether this effect is impacted by environment would require evaluation in a different geographical zone.

IH is classically a diagnosis of exclusion and it is imperative that serious underlying causes are not missed. Of the 408 cases initially coded as TS/IH, 44 (10.8%) did not have a final diagnosis of IH. Due to coding limitations, 12 (25%) patients were diagnosed with transient synovitis of other joints, which could not be coded separately in the system. Documented normal examination (n = 3) may represent mis-coding and six children were yet to have a final diagnosis determined at the time of data collection. Therefore, 23 (5.6%) of children coded as TS/IH had alternative diagnoses for their symptomatology. The final diagnosis for nine of these patients was benign and would not require speciality referral [soft tissue injury (n = 6), benign growing pains of childhood (n = 1), non-specific abdominal pain (n = 1), Osgood-Schlatter disease (n = 1)]. SA is a serious underlying cause of hip irritability that requires emergency treatment and its misdiagnosis can have disastrous sequelae. None of the patients in our cohort initially diagnosed as IH had SA, though one patient initially thought to have SA was finally diagnosed as IH. It remains a reservation of our study that longer-term follow-up is not (as yet) available for this cohort, in particular, to look for subsequent Perthes condition.

Of the patients who were finally diagnosed with IH (n = 354), the investigation rate was low, with 255 (72%) of patients not having any investigations on initial presentation. This supports the notion that investigative procedures for IH can be selective, based on clinical suspicion with rarer conditions declaring themselves in time due to persistent symptomatology [14]. This contrasts to historical cohorts in which alternative diagnoses were ruled out through investigations, before IH was diagnosed. This indicates that patients with benign symptomatology can be safely managed in the ED, limiting the number of unnecessary investigations (and usually obviating the need for ultrasound) and speciality referrals.

## Conclusions

In summary, the key findings of this one-year descriptive epidemiological study are that IH in this locale has: (i) an atypical age profile (age distribution shift to younger), (ii) no marked association with social deprivation (in contrast to other studies), and (iii) a ‘spring preponderance’. We suggest that most cases can safely be managed in the ED without recourse to further investigations or speciality referral.

## Appendices

**Table 2:** Diagnosis codes.

Diagnoses	Codes
Transient synovitis/irritable hip	M67.3
Pain in joint (nontraumatic)	M25.5
Pain in limb (nontraumatic)	M79.6
Juvenile osteochondrosis of pelvis	M91.0
Juvenile osteochondrosis of head of femur, Legg-Calve-Perthes	M91.1
Juvenile osteochondrosis of hip and pelvis – Coxa plana	M91.2
Juvenile osteochondrosis of hip and pelvis – Pseudocoxalgia	M91.3
Other juvenile osteochondrosis of hip and pelvis	M91.8
Juvenile osteochondrosis of hip and pelvis, unspecified	M91.9
Slipped upper femoral epiphysis (nontraumatic)	M93.0
Reactive arthropathy, unspecified	M02.9
Other reactive arthropathy	M02.8
Juvenile rheumatoid arthritis	M08.0
Juvenile arthritis	M08.8
Monoarthritis, NEC	M13.1
